# Fatal enteric plexus neuropathy after one dose of ipilimumab plus nivolumab: a case report

**DOI:** 10.1186/s40425-018-0396-9

**Published:** 2018-08-31

**Authors:** Jacob Appelbaum, David Wells, Joseph B. Hiatt, Gideon Steinbach, F. Marc Stewart, Hannah Thomas, Paul Nghiem, Raj P. Kapur, John A. Thompson, Shailender Bhatia

**Affiliations:** 1 University of Washington, Seattle, WA USA; 2 Fred Hutchinson Cancer Research Center, Seattle, WA USA; 3 Seattle Children’s Hospital, Seattle, WA USA; 4 Medical Oncology, Seattle Cancer Care Alliance, 825 Eastlake Ave E., Mailstop # CE2-102, Seattle, WA 98109 USA

**Keywords:** Ileus, Merkel cell, Ipilimumab, Nivolumab, Immune-related adverse events, Pseudo-obstruction, Enteric neuropathy, Myenteric plexopathy, Neuritis, Immune checkpoint inhibitor

## Abstract

**Background:**

Immune checkpoint inhibitors (ICIs) are the treatment of choice for several cancers and can be associated with remarkable clinical benefit, but can also cause serious immune-related adverse events (irAEs). Management of rare and severe irAEs is challenged by an incomplete knowledge of their natural history and pathogenetic mechanisms. We report a case of fatal acute-onset gastro-intestinal (GI) hypomotility from myenteric plexus neuropathy following a single dose of ipilimumab plus nivolumab given for treatment of Merkel cell carcinoma (MCC).

**Case presentation:**

A 66-year-old man with recurrent metastatic MCC involving several organs (liver, bones and disseminated retroperitoneal lymphadenopathy) developed profound pharyngeal dysphagia and ileus that started 7 days after receiving a single administration of combination immune checkpoint blockade consisting of nivolumab (3 mg/kg) and low-dose ipilimumab (1 mg/kg). A swallowing study showed oropharyngeal dysfunction and aspiration. Imaging studies were consistent with diffuse intestinal paresis. An extensive work-up did not reveal obvious causes of these symptoms, and enteric plexopathy was suspected. Empiric immune suppressive therapy was initiated urgently. Despite an escalating immunosuppressive regimen that included high dose steroids, tacrolimus and therapeutic plasma exchange, no improvement in GI motility was seen and the patient suffered repeated episodes of aspiration. Seven weeks after the onset of GI hypomotility, the patient succumbed to sepsis from intestinal perforations. At autopsy, histologic specimens obtained from the entire GI tract (pharynx to rectum) showed near complete loss of ganglion cells within the myenteric and submucosal plexuses. An associated inflammatory infiltrate was not seen, suggesting a ‘burned out’ phase of illness. C4d complement deposition was found at the ganglionic sites, suggesting antibody-mediated pathogenesis. Remarkably, at sites of previously suspected Merkel cell metastases, no residual viable Merkel cell carcinoma was identified.

**Conclusions:**

GI-tract paresis due to myenteric neuritis is a rarely reported toxicity of ICIs. Because the window of reversibility is likely to be very brief, quick and decisive interventions are warranted. Subtle functional and anatomic perturbations of the myenteric nervous system from the use of ICIs may be more prevalent than realized and should be suspected and addressed in both clinical and investigational settings.

## Background

Immune checkpoint inhibitors (ICIs) have been associated with unprecedented clinical success in patients with many cancer types and have ushered in a therapeutic revolution in cancer immunotherapy. Monoclonal antibodies of this class stimulate T cell function by blocking suppressive ‘checkpoint’ receptors such as cytotoxic T-lymphocyte antigen (CTLA)-4 (e.g. ipilimumab), programmed death (PD)-1 (e.g. pembrolizumab, nivolumab) or its target ligand, PD-L1 (avelumab, atezolizumab, durvalumab) [1]. ICIs are now recommended as initial or adjuvant treatments for non-small cell lung, kidney, Merkel cell, melanoma, and urothelial cancers. ICIs are also being widely explored for recurrent or refractory metastatic cancers, and may be used in any advanced cancer with a mismatch repair deficiency [2].

While ICIs have substantially improved cancer outcomes, inhibiting immune regulatory mechanisms can also result in unique toxicities, termed immune related adverse events (irAEs), which include, but are not limited to, colitis, hepatitis, dermatitis, pneumonitis and endocrinopathies such as hypophysitis [3, 4]. The inflammatory conditions brought on by immunotherapy are varied and unpredictable [5, 6]. The risk of occurrence, severity, and timing of onset of irAEs may depend on the agents used and dose-levels. The rate of irAEs identified as grade 3 or higher has varied from 5 to 26% (pembrolizumab [7–9], nivolumab [10, 11]), 15–27% (ipilimumab at 3 mg/kg [12, 13]), 18–34% (ipilimumab at 10 mg/kg [12, 14]) to as high as 55% (ipilimumab plus nivolumab [13, 15] or ipilimumab plus dacarbazine [16]). Management of clinically significant irAEs begins with discontinuation of ICI treatment and initiation of corticosteroids, typically prednisone at a dose of 1 to 2 mg/kg or equivalent [6]. Increasing corticosteroid dose and/or addition of other immunosuppressive agents, such as calcineurin inhibitors, purine antagonists (including mycophenolate and azathioprine), or tumor necrosis factor (TNF) alpha inhibitors, is usually effective in controlling irAEs [17]. Close vigilance, prompt identification and treatment of irAEs are essential to prevent potentially life-threatening complications and long-term morbidity.

As experience with the ICIs grows, rare and sometimes fatal side effects are emerging, including myocarditis [18], asystole, encephalitis, aphasia, a parkinsonoid syndrome and various ocular inflammatory syndromes, all of which are likely due to unwanted immune stimulation in critical organs following immune checkpoint therapy [19]. Because of the rarity, unexpected nature, unpredictable clinical course, and varied anatomic locations of these irAEs, shared pathogenetic mechanisms underlying these toxicities can be difficult to discern. Also, other less severe (and possibly more frequent) manifestations of immune checkpoint blockade may not attract attention and pass unrecognized as immunotherapy-mediated.

We had previously reported the case of a patient with metastatic melanoma who developed severe constipation from myenteric ganglionitis following treatment with two doses of ipilimumab [20]. We now report a case of a patient with metastatic Merkel cell carcinoma (MCC) who developed severe pharyngeal dysphagia and severe intestinal paresis associated with diffuse loss of myenteric and submucosal ganglia following one dose of combination checkpoint blockade with ipilimumab plus nivolumab. These two cases involving two different cancer types together suggest a syndrome of ICI-induced intestinal paresis caused by immune-mediated destruction of the myenteric plexus.

## Case presentation

A 66-year-old Caucasian man presented with recurrent, metastatic MCC that involved several sites in liver, bones and disseminated lymphadenopathy. Primary MCC diagnosed 3 years before was located on the forearm without lymph node involvement and was treated with surgery and adjuvant radiation therapy. Serologic testing for antibodies to the Merkel cell polyomavirus T-antigen oncoprotein was negative [21]. The patient’s past medical history included hypertension, hyperlipidemia, coronary artery disease treated with stenting, gastroesophageal reflux, and mild cerebral palsy that had been stable. His activities of daily living were not limited by his comorbidities. Medications included lisinopril, simvastatin, aspirin, omeprazole, and zolpidem. His ECOG performance status was 0 and his physical exam revealed a stable speech impediment. After a discussion of various systemic therapeutic options for his metastatic MCC, the patient decided to participate in a clinical trial investigating ICIs in virus-associated cancers including MCC (clinicaltrials.gov; NCT02488759). The patient provided informed consent for treatment and for information sharing as part of a research protocol approved by the University of Washington/Fred Hutchinson Cancer Research Center IRB. He received dual immune checkpoint blockade with one dose of ipilimumab (1 mg/kg) plus nivolumab (3 mg/kg) administered on day 1.

At the time of treatment on day 1, he had no concerning neurologic, GI or other symptoms. Seven days after the administration of first dose of combination immunotherapy, the patient reported diffuse muscle aches, urinary retention, abdominal distention and a sensation of ‘gagging’ with swallowing attempts. He began taking low doses of oxycodone 5 mg every 4-6 h with partial relief. Four days later (day 11 post-immunotherapy), he was hospitalized due to worsening symptoms and a need for supportive care. A CT scan showed air-filled loops of small and large bowel and an increased stool burden within the right colon consistent with pseudo-obstruction without clear signs of mechanical obstruction. Diverticulosis was present without pericolonic fat stranding suggestive of diverticulitis. Despite methylnaltrexone (given for treatment of possible opioid induced constipation) and escalating doses of laxatives, no bowel movement was produced. Abdominal distention and constipation persisted, together with recurrent bouts of emesis. On day 13 post-immunotherapy, the patient developed hypoxic respiratory failure, attributed to an episode of aspiration, and required mechanical ventilation. A CT scan showed mildly distended fluid filled small bowel loops, moderate to large stool burden in the right colon and fecalization of the terminal ileum contents. Microbiological blood and urine cultures were sterile. The patient was treated with supportive care for 5 days after which extubation was successful. Video laryngoscopy showed a widely patent airway, with severe aspiration due to swallow delay/discoordination, weakness of base of tongue, hyolaryngeal excursion, moderate to severely weak pharyngeal constrictors and cricopharyngeal dysfunction. The patient was also noted to have nasal regurgitation due to uncoordinated velar elevation. A fluoroscopic study of oral contrast (gastrografin) showed absence of contrast progression to colon 48 h following administration.

On day 22 post-immunotherapy, the patient’s respiratory status decompensated again and repeat intubation was required. A second CT scan of the abdomen showed partial regression of the MCC metastases in the liver and resolution of retroperitoneal lymphadenopathy. A fluid-filled esophagus and dilated loops of proximal small bowel, again without a transition point, was essentially unchanged from the prior scan. Placement of a nasogastric tube returned nearly 2 L of bilious and later feculent material. This clinical presentation of diffuse intestinal paresis and pharyngeal dysfunction following administration of immune checkpoint blockade suggested the possibility of immune-mediated neuropathy of the enteric nervous system or a similar irAE (based on our prior experience with a similar case published by our group) [20]. Due to this concern, treatment with methylprednisolone 2 mg/kg daily was initiated on day 22 post-immunotherapy.

Evaluating whether this patient was suffering from an immune adverse effect of checkpoint immunotherapy on myenteric ganglia was challenging because the myenteric plexus, which lies within the muscularis propria, is evaluable only by a full-thickness biopsy. The feasibility of this endoscopic procedure was discussed extensively with the multidisciplinary team involved in the patient’s care, but was considered to pose a prohibitively high risk for perforation and poor wound healing. There was also concern that use of endoscopic cautery after biopsy could ignite bowel gases in the setting of obstipation. The patient’s three prior episodes of pulmonary decompensation resulted in increased risk for prolonged intubation following anesthesia. These considerations also placed the patient at prohibitively high risk for surgery. Thus, invasive diagnostic testing was not attempted. Serologic testing for antibodies to the acetylcholine receptor was negative. Additional serologic tests for anti-Hu antibodies as well as antibodies to P/Q type and N type calcium channels, neuronal (V-G) potassium channels, striated muscle, antineuronal nuclear antibodies, anti-glial nuclear antibodies, Purkinje cell cytoplasmic antibodies, amphiphysin antibodies and CRMP 5 were negative. The patient did not exhibit any arrhythmia during periods of cardiac monitoring.

**Fig. 1:**
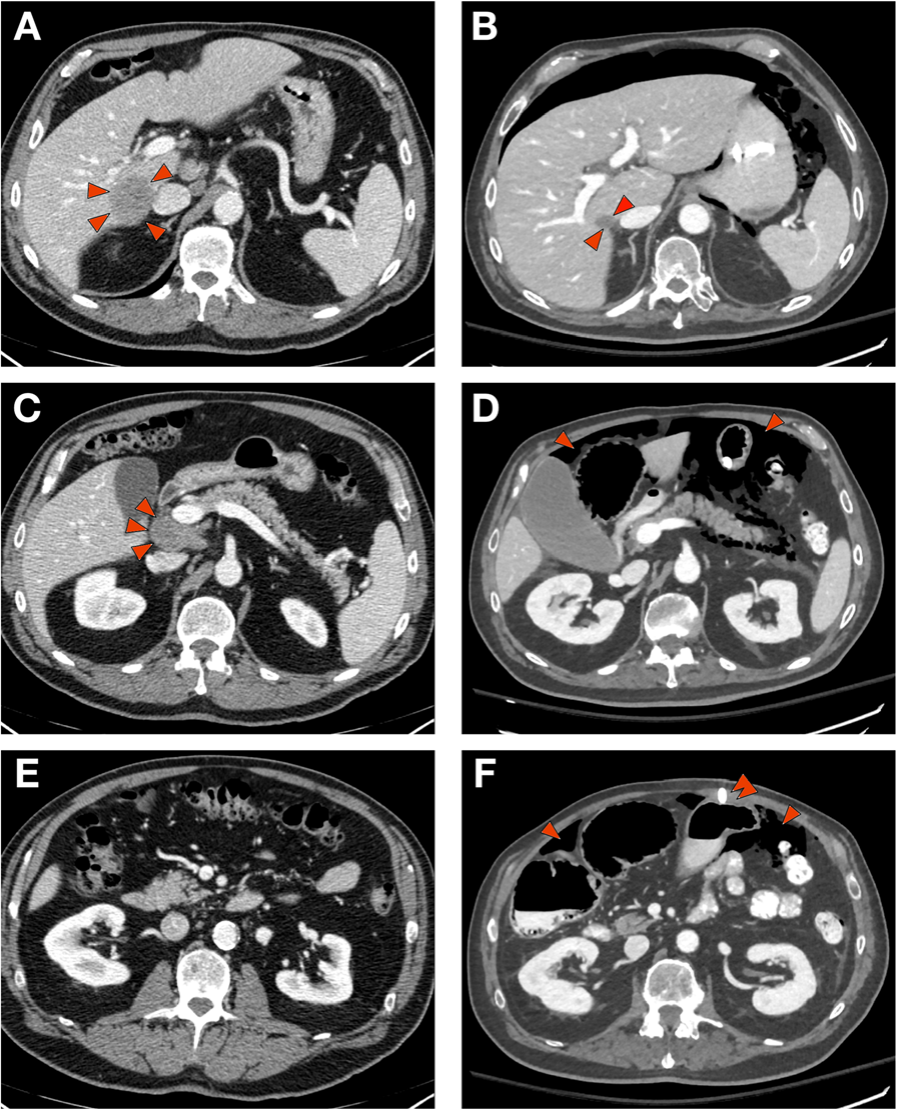
CT images obtained following the administration of IV contrast prior to (panels **a**, **c**, and **e**) and 51 day following treatment (panels **b**, **d**, **f**) with ipilimumab and nivolumab. The pretreatment scans show a 4.2 × 4.0 cm lesion within the right lobe of the liver lateral to the inferior vena cava (**a**, the lesion is identified by arrowheads), as well as peripancreatic retroperitoneal adenopathy (**c**, identified by arrowheads). Following treatment, the lesion within the liver has decreased to 2.0 × 1.6 cm (**b**, identified by arrowheads) and the retroperitoneal adenopathy has resolved (**d**). Dilated loops of large bowel as well as peritoneal air is present (panels **d** and **f**, identified by arrowheads) as well as a percutaneous gastrostomy tube (panel **d**, double arrowhead)

Following 1 week of treatment with high dose steroids, there was no apparent improvement in gut motility and the patient continued to have no bowel movement or passage of flatus. On day 30, the patient was started on tacrolimus with a target trough of 5–15 ng/mL. Enemas were attempted to induce defecation, but were unsuccessful. Plasmapheresis to remove potential pathogenic autoantibodies was initiated on day 45 post-treatment and repeated every other day for a total of 5 treatments. A venting gastrostomy tube was placed on day 49 due to worsening abdominal distention. On day 51 post-treatment, a CT scan showed focal inflammatory stranding and air involving the transverse colon compatible with diverticulitis with contained perforation. The known MCC tumors had regressed further (see Fig. 1). The percutaneous gastrostomy tube was noted in the appropriate position. Surgical consultants suggested that repairing the perforation was very high risk and would not correct the underlying suspected pathology. Therefore, best supportive care was continued for a further 5 days. Following absence of improvement, the patient and family decided to transition to comfort care. On day 58 post-treatment, the patient died from progressive sepsis complicated by multi-organ failure.

**Fig. 2:**
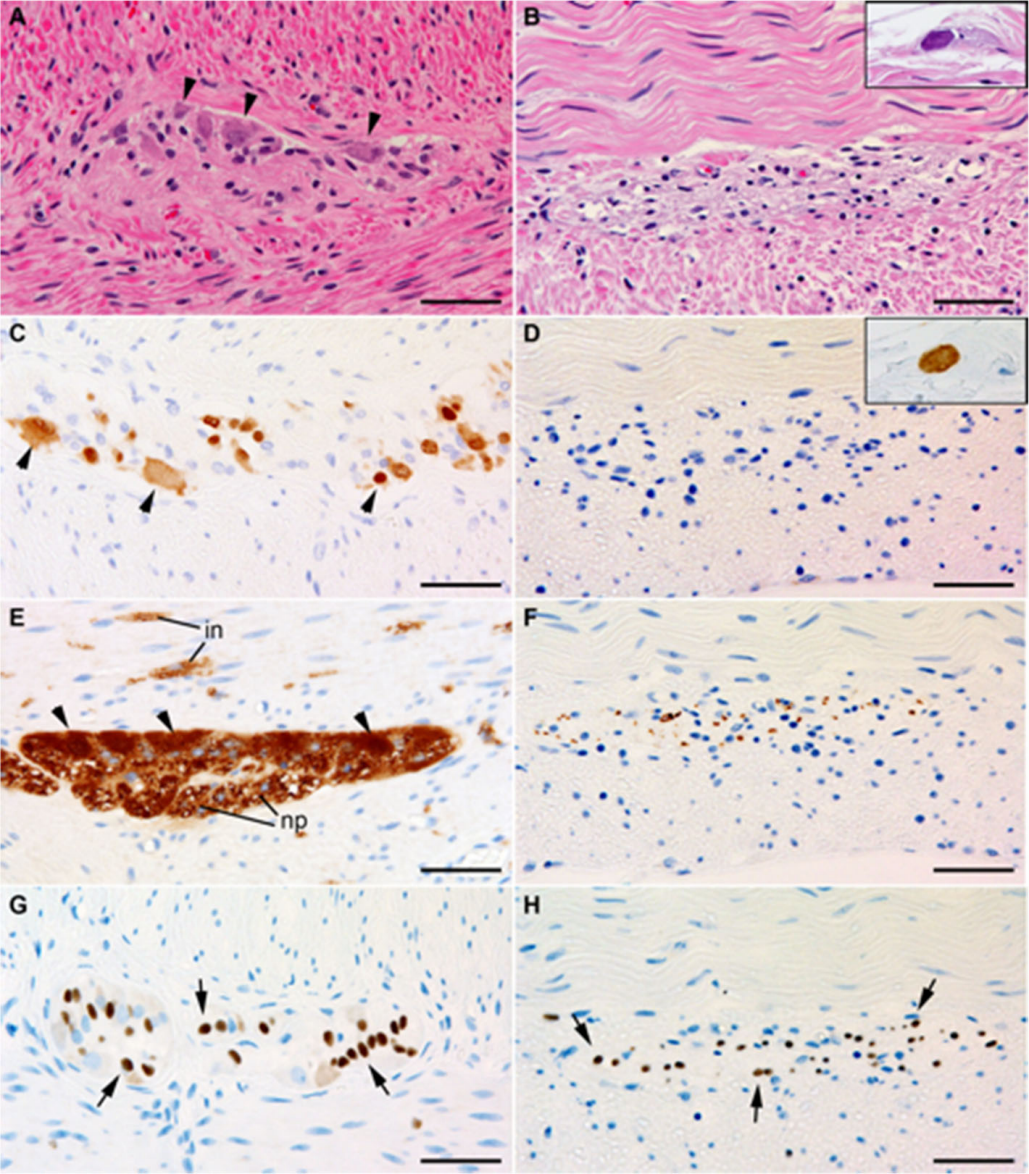
Representative histology from a control section of comparably autolyzed colon (not from this patient) demonstrating a representative myenteric ganglion (**a**) containing ganglion cells (arrowheads) and scattered surrounding glia. This is in contrast to the patient’s ganglia (**b**) which show morphologic absence of ganglion cell bodies and no conspicuous inflammatory infiltrate. While myenteric ganglions cells in control tissue (not from this patient) are highlighted by anti-Hu (**c**, arrowheads), no immunoreactive ganglion cells are present in this patient (**d**). Rare submucosal ganglion cells were seen (Fig. 2b and d, insets). Similarly, immunohistochemistry using PGP9.5 on the control myenteric plexus (**e**) highlights neuronal bodies (arrowheads), adjacent neural processes (np) and intramuscular nerves (in), in contrast to the patient whose myenteric plexus (**f**) demonstrates sparse residual processes in the plexus and no intramuscular nerves. Immunohistochemistry for SOX10 in both the control plexus (**g**) and the patient’s tissue (**h**) show residual glial cells (arrowheads). Scale bars: 50 μm

After obtaining permission from the family, an autopsy was performed. Findings upon gross examination included peritoneal fluid contaminated by stool and diverticulosis with two associated perforations. The spleen was enlarged with diffluent parenchyma, and the liver histology showed cholestasis and biliary pools, findings associated with sepsis. Extensive sampling of the gastrointestinal tract was performed to include esophagus, stomach, small and large bowel, with microscopic evaluation revealing severe hypoganglionosis along its entire length, with absence of ganglion cells within the myenteric plexus and only rare submucosal ganglion cells (see Fig. 2a-h). Within the myenteric plexus residual glial cells remained, however no conspicuous lymphocytic infiltrate was identified. Immunohistochemistry with appropriate controls was performed. Within the myenteric plexus, no immunoreactivity for neuron-specific anti-Hu antibodies was seen, consistent with complete absence of ganglion cells. Furthermore, while staining with antibody PFP9.5 demonstrated scattered residual neuronal processes, likely the projections from extra-enteric autonomic or sensory ganglia, no staining of myenteric ganglion cell bodies or intramuscular nerves was seen, again supporting extensive loss of ganglion cells within the myenteric plexus. Rare submucosal ganglion cells were identified with each neuronal marker (Fig. 2b and d, inset), but were far fewer than normal. Immunostaining for SOX10 highlighted condensation of residual glial cells consistent with the death of nearby nerve cells [22]. A trichrome stain demonstrated some fibrosis in the areas of residual glial cells, consistent with a chronic injury response (not shown). A search for other sites of injury revealed reduced numbers of nerves and ganglia in the urinary bladder and epicardium as well (not shown).

The area of radiologic abnormality associated with clinical suspicion for residual MCC within the right lobe of the liver was grossly identified as a pale-yellow discolored nodule within the hepatic parenchyma. Microscopic examination demonstrated fibrosis and necrosis without identifiable residual carcinoma. Representative lymph nodes in the areas of previous radiologic enlargement were similarly free of carcinoma by gross inspection as well as histologic examination (not shown).

**Fig. 3:**
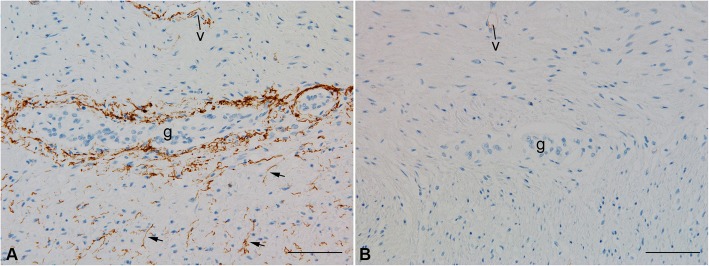
C4d immunoreactivity around ganglia and nerves. **a** Intense C4d immunolabelling is observed around an aneuronal ganglion (g) and in the location of small intramuscular nerves (arrows) in the colon of the patient. **b** In contrast, no significant C4d expression is observed in ganglia (g) in the colon from a cadaveric control patient with no history of ipilimumab or novilumab exposure or dysmotility. v, blood vessel. Scale bars = 100 μm

To investigate the possible pathogenetic involvement of the humoral immune system, we stained histologic sections of colon tissue for C4d. C4d is a serine protease in the classical complement cascade that can be generated and deposited during complement activation by the antibody complexes, resulting in formation of multi-molecular complexes with immune effector and cytolytic activity [23]. We found intense C4d immunoreactivity around and occasionally within the aneuronal ganglia of our patient (Fig. 3a), as well as in vessel walls and along probable intramuscular nerves in the muscularis propria. The perineuroglial pattern was observed around the patient’s myenteric plexus, but not around his residual submucosal nerves. No immunoreactivity was observed in sections for which the primary antibody was omitted. In contrast, sections from the colons of 4 controls (cadaveric samples with similar autolysis, but no history or intestinal dysmotility) showed only very weak immunolabelling of some vascular endothelial surfaces and no perineural C4d immunoreactivity (Fig. 3b). These results demonstrate local complement activation at the site of organ dysfunction (the myenteric plexus) and support a possible contributing role of antibodies in the pathogenesis of this patient’s myenteric plexopathy.

## Discussion and conclusions

Immunotherapy with ICIs improves cancer outcomes, but can be associated with varied side effects. In the case above, a single dose of nivolumab plus ipilimumab led to complete eradication of a patient’s widely metastatic MCC, but unfortunately was complicated by rapid and irreversible destruction of the patient’s myenteric ganglions that ultimately proved fatal. The case described above illustrates both the promises and pitfalls of the power of immune checkpoint blockade.

The striking clinical findings in this case were a marked impairment in managing oral secretions, a lack of flatus/bowel movements, uncoordinated swallowing seen on video laryngoscopy, and radiologic findings of fluid-filled bowels with minimal transit of oral contrast. These symptoms began within 1 week after the initiation of immune checkpoint blockade and prior to the use of opiates and several of the other therapies, including the use of enemas, tacrolimus, methylnaltrexone, and prednisone, which were applied during his hospitalization and have been potentially associated with gastrointestinal perforation [24, 25].

The striking absence of bowel movements and atony of the gut in this patient suggested damage to the myenteric plexus, similar to a case described previously by our group [20], in which a patient had developed severe constipation 14 days after receiving high dose ipilimumab (10 mg/kg every 3 weeks for 3 doses). The previously described patient had succumbed to an unrelated cardiac event that occurred 42 days following ipilimumab initiation, and the autopsy revealed a dense peri-neural T-cell infiltrate surrounding neurons and disrupting nerve fibers within the myenteric plexus. He had not received any immune suppressive therapy, as constipation was not yet recognized as a potential irAE. Our current case shares several similarities with the previous case, but also has several contrasting features:

1. In the current case, no conspicuous inflammatory infiltrate was seen around the ganglia at the time of autopsy (58 days post-treatment). This absence of infiltrating inflammatory cells in the ganglionic region could be due to either a ‘burned-out’ phase of the illness wherein the target of the immune attack has already been destroyed, or due to intense escalating immune suppression, or both.
2. The present case involved the entire length of the gastrointestinal tract (pharynx to rectum) both clinically and pathologically, whereas in the previously reported case, constipation had predominated and the patient did not report any complaints related to swallowing. Despite these differences in clinical presentation, we now performed a careful retrospective review of autopsy slides from the prior case that revealed lymphocytes infiltrating myenteric ganglia throughout the gastrointestinal tract (data not shown).
3. Both cases were associated with quick-onset immune responses against the cancer, resulting in complete pathologic eradication of disseminated metastases. Even though the two patients had differing cancer types (melanoma and MCC), there remains the possibility of a shared antigen between the myenteric neurons and the cancer cells.

Two other instances of intestinal pseudo-obstruction associated with immune checkpoint inhibitor therapy have been recently reported, but the myenteric plexus was not evaluated in these cases. A 57-year-old man with poorly differentiated neuroendocrine tumor developed pseudo-obstruction after 11 doses of pembrolizumab [26]. In this case, mucosal biopsies showed chronic inflammation, but deep biopsies were not obtained. The patient’s condition was refractory to corticosteroids, and ultimately proved fatal when intestinal perforation lead to multisystem organ failure. A 62-year-old man with non-small cell lung adenocarcinoma developed pseudo-obstruction following 14 doses of nivolumab (3 mg/kg) [27]. Prednisone (2 mg/kg/day) was initiated 7 days after the onset of symptoms and led to resolution of symptoms within 6 days of treatment. The corticosteroids were tapered over 30-day period, and at the time of the case report, the patient has remained without recurrence of his cancer or his gastrointestinal irAE for 6 months. Because neither of these reports evaluated the myenteric plexus, limited distinctions between fatal and non-fatal cases of ICI-associated pseudo-obstruction can be made.

The full spectrum of neurologic complications from checkpoint inhibitors is unknown, but is likely to be broad and heterogeneous. Others have reported cases of Guillain-Barré-like demyelinating polyneuropathy induced by ipilimumab, but no autopsy was available [28]. Patients with melanoma and MCC could be hypothesized to be at increased risk for development of neuropathy when treated with immune checkpoint blockade, possibly due to shared tumor and neuronal expression of gangliosides [29], Hu [30], or other antigens [31], reflecting the shared embryological origin (neural crest) of melanocytes, Merkel cells and neurons (including neurons of the enteric plexus). T cells activated by ICIs could mediate direct cytotoxic effects on both neurons and tumor cells due to shared expression of major histocompatablity complex (MHC)-restricted peptide epitopes. Alternatively, humoral immune mechanisms involving deposition of antibody complexes to shared epitopes and subsequent complement recruitment/activation may be primarily responsible, as suggested by the finding of C4d deposition around the myenteric plexus. Unfortunately, due to a lack of serial blood and tissue samples collected prior to and during the course of this toxicity, we could not perform additional analyses to investigate the culprit antigens in this patient. We urge investigators and institutions to establish mechanisms for systematic serial collections of blood and tumor samples, beyond clinical trials, at baseline prior to initiating ICIs and at the time of presentation of interesting clinical situations (such as unusual response pattern, progression and/or toxicity). Such efforts can significantly accelerate the discovery of mechanisms and predictive biomarkers for both response and toxicity with ICIs.

The focal activation of complement proteins surrounding the myenteric plexus suggests the presence of antibodies directed at antigens expressed on myenteric nervous system. While our patient tested negative for anti-Hu antibodies, the development of anti-Hu antibodies is one well described mechanism of paraneoplastic neuropathy associated with both small cell lung cancer [32] and Merkel cell carcinoma [33]. Whether anti-Hu antibodies are directly toxic to neurons is unclear [34], but their presence is associated with sera that damages neurons directly in vitro [35]. A case of idiopathic myenteric ganglionitis associated with the presence of anti-Hu antibodies in a patient without a known cancer diagnosis improved coincidently with a decrease in anti-Hu antibody titer following treatment with steroids [36]. The overall frequency of anti-Hu or other anti-neuronal antibodies in patients with Merkel cell carcinoma without paraneoplastic syndromes has not been investigated. In addition, the role of pathogenic autoantibodies as etiologic agents for constipation unrelated to immunotherapy is also unknown [37, 38]. Antibody-mediated mechanisms have also been suggested in other irAEs. An intriguing report found that expression of CTLA-4 within pituitary neurons led to formation of CTLA-4/ipilimumab immune complexes, triggering complement activation and hypophysitis in a murine model [39]. Extending this finding, the authors performed a retrospective review of 7 patients who had developed hypophysitis after receiving ipilimumab for the treatment of melanoma and found that all seven had developed anti-pituitary antibodies, whereas pretreatment samples as well as samples from 13 control samples did not contain anti-pituitary antibodies.

In our patient, the paucity of inflammatory cells and absence of neurons within the gut at autopsy suggests that further intensification of the patient’s immunosuppression closer to the time of death would not have been beneficial. This observation strongly suggests careful consideration of early and intensive immunosuppression, soon after development of suggestive symptoms, ideally after rapid confirmation of a pathologic diagnosis. While immune suppression was escalated fairly rapidly in our case after day 22, the initial delay in beginning steroids and the late introduction of plasmapheresis (day 45) may have contributed to irreversible damage. Until the exact mechanisms of this unusual and severe toxicity become apparent, we suggest prompt institution of therapies targeting both cellular and humoral immunity, the latter probably being quite important given the finding of C4d deposition at the target site of myenteric plexus. Concurrent rather than sequential implementation of immune suppression should be considered. Analysis of pooled data from Phase 2 and 3 trials of immunotherapy with combined ipilimumab plus nivolumab shows that antitumor efficacy was similar between those patients who discontinued immunotherapy due to irAEs and those who did not, suggesting that early discontinuation of ICIs and institution of immunosuppression may not completely abrogate anti-cancer activity [40].

Because of uncertainty regarding the effects of immune suppression on ICI-induced anti-tumor response, a prompt and definitive pathologic diagnosis of ganglionitis may be needed to justify aggressive immunosuppresion. In our case, the pathologic mechanism for the clinical hypomotility syndrome was suspected relatively early on, but still could not be confirmed prior to death because the process of obtaining a full thickness biopsy was evaluated as prohibitively high risk. Obtaining tissue for histologic diagnosis should be considered early in the clinical course with input from GI and surgery teams, especially when the clinical diagnosis is uncertain. Sigmoidoscopy to the splenic flexure with extensive biopsies is usually sufficient for the diagnosis of immune mediated colitis. In the absence of diarrhea an esophagogastroduodenoscopy and sigmoidoscopy or colonoscopy with biopsies may be useful in evaluating for enteritis and signs of intestinal paresis. Deep rectal biopsies can safely evaluate Meissner’s submucosal plexus for neuropathy. Full thickness biopsies, usually performed by a surgical team, involve the entire thickness of the colon, which carries additional risks. Surgical biopsy may be reserved for cases with an unclear diagnosis following the above evaluations. In addition to establishing histologic diagnosis, there is also a need for objectively monitoring functional improvement in gut motility, especially to guide management of immune suppressive therapies. Potential cases could be evaluated by CT scans and by monitoring contrast transit time as well as video fluoroscopic swallow studies and speech pathology evaluation. Rectal or esophageal manometry may be helpful in objectively tracking progress in gut motility.

The rarity of reported myenteric plexopathy as a potential irAE highlights the need to disseminate experiences such as this one, so that early recognition may prompt intervention. While constipation is a frequently reported adverse reaction in trials of immunotherapy, this has not typically been regarded as an irAE. It is unclear if neuronal injury commonly contributes to immunotherapy-related constipation with a mild presentation [37, 38], or if it is a rare phenomenon that tends to be severe. We suggest paying close attention to hypomotility symptoms in the clinical and investigational settings and having a high index of suspicion for potential myenteric neuropathy, as there is a potential for progressive, severe, and likely irreversible injury if myenteric inflammation is left unchecked. Identifying severe cases prior to the ‘burned out’ phase will be required to facilitate any potential benefit from immunosuppression.

## Abbreviations

(CTLA)-4: cytotoxic T-lymphocyte antigen
(irAEs): Immune related adverse events
(MCC): Merkel cell carcinoma
(PD-1): Programmed-death 1
(PD-L1): Programmed death ligand 1
